# Antioxidants and Quality Changes of Thermally Processed Purple Corn (*Zea mays* L.) Milk Fortified with Low Sucrose Content during Cold Storage

**DOI:** 10.3390/foods12020277

**Published:** 2023-01-06

**Authors:** Khursheed Ahmad Shiekh, Thitirat Luanglaor, Natchaya Hanprerakriengkrai, Saeid Jafari, Isaya Kijpatanasilp, Nicha Asadatorn, Randy W. Worobo, Alaa El-Din Ahmed Bekhit, Kitipong Assatarakul

**Affiliations:** 1 Department of Food Technology, Faculty of Science, Chulalongkorn University, Bangkok 10330, Thailand; 2 School of Agro-Industry, Mae Fah Luang University, Thasud, Chiang Rai 57100, Thailand; 3 International Programme in Hazardous Substance and Environmental Management (IP-HSM), Graduate School, Chulalongkorn University, Bangkok 10330, Thailand; 4 Department of Food Science, College of Agriculture and Life Sciences, Cornell University, Ithaca, NY 14853-5701, USA; 5 Department of Food Science, University of Otago, Dunedin 9054, New Zealand

**Keywords:** anthocyanins, antioxidant activity, purple corn milk, refrigerated storage, sensory acceptance, shelf-life, thermal processes

## Abstract

Purple corn kernels were subjected to boiling and steaming times of 5–15 min to extract purple corn milk (PCM). Pasteurized and unpasteurized PCM samples were investigated for changes in anthocyanins, antioxidants, and physicochemical properties. Anthocyanins, total phenolics, antioxidant activity, color and viscosity values showed promising results in pasteurized PCM samples extracted from kernels steamed for 5 min (PPCM-S5) compared to other samples (*p* ≤ 0.05). Changes in L*, a* and b* values, total phenolics and DPPH activity were lowered in PPCM-S5 samples with higher retention of anthocyanins compared to the PCM extracted from boiled kernels (*p* ≤ 0.05). PCM extracted from 5 min steamed kernels fortified with 4% sucrose (PCM5-S4) after pasteurization revealed the lowest changes in color, pH, total soluble solid and viscosity during 12 days of storage at 4 °C compared to the unpasteurized PCM without sucrose and pasteurized PCM fortified with 6% sucrose. Additionally, pasteurized PCM5-S4 samples marked the highest anthocyanins, total phenolics and antioxidant activity during storage. Microbial load was lowest in pasteurized PCM5-S4 samples stored at 4 °C for 12 days. However, coliforms, yeast or mold and *Escherichia coli* were not present in the thermally processed PCM samples. The highest sensory scores were obtained in PCM5-S4 at day 12 of storage compared to PCM without any treatment. Therefore, pasteurized PCM extracted from 5 min steamed purple corn kernels retained bioactivity along with 4% sucrose fortification resulted in higher sensory acceptability. As a consequence the shelf-life of PCM5-S4 sample was extended up to 12 days at 4 °C.

## 1. Introduction

The functional value of foods and beverages is of prime importance for consumers to balance their nutritional requirements and to reduce the risk of chronic diseases [1]. Chemical-additive-free beverages from different fruits and vegetables with minimal processing are highly recommended in supermarkets worldwide. Color diversity of plant-based products such as fruits, vegetables and fresh kernel foods including corn cultivars are abundant sources of different phytochemicals such as anthocyanins, carotenoids and conjugated polyphenols with enormous bioactivity and nutraceutical benefits [2]. The functional components in fresh foods or beverages such as vitamins, minerals, antioxidants, dietary fiber, phytosterols and essential fatty acids play a vital role in regulating normal biological activity in the human body [3]. Additionally, vitamins and other antioxidants balance redox reactions by quenching the free radical pro-oxidants responsible for DNA damage or membrane degradation, thereby decelerating cellular aging [4,5]. Plant-based products contain naturally synthesized medicines that have health-promoting properties [6].

During the past decade, the processing of anthocyanin-rich foods without chemical additives has gained attention due to their potential antioxidant, antimicrobial, anti-inflammatory, anticancer, antidiabetic and vision-improving benefits [7]. Purple corn (*Zea mays* L.), a native cultivar of Peru, has been used for the preparation of traditional beverages, desserts and colorants for food applications [8]. Purple corn is consumed in Thailand as steamed or boiled due to its appealing food quality and health-promoting properties [9]. Maize and soybean are also produced in a sustainable and ecofriendly way to ensure feed sufficiency at lower nitrogen and carbon footprints in China [10]. Purple corn is an abundant source of anthocyanins such as peonidin-3-O-glucoside, cyanidin-3-O-glucoside and pelargonidin-3-O-glucoside [11]. Anthocyanins from purple corn have been fortified in milk to evaluate their efficacy in retarding the oxidation of unsaturated fatty acids [12]. Nevertheless, the functional value of anthocyanins and other essential compounds during processing should be safeguarded by the implementation of optimum thermal-processing-unit operations to ensure safe storage quality. 

Conventional boiling, steaming and pasteurization processes have been assessed to monitor the changes in physiochemical properties of foods and beverages. Thermal processes employed in wheat flour at varying temperature ranges upon interaction with water molecules modify the behavior of amylose and amylopectin to induce gelatinization [13]. Of these thermal processes, steaming was more effective at preserving the functional properties of green beans with improved food safety [14]. The highest content of polyphenols and antioxidant activities were retained in the pre-steamed leaves used for extraction of wild rocket juice [15]. Additionally, steaming of frozen vegetables increased the total antioxidant activity and bio-accessibility of polyphenols compared to boiling leading to the degradation of polyphenols and decreased antioxidant capacity [16]. The microbial quality of beverages including bacterial, yeast and mold growth have been delayed by the pasteurization treatment of beverages to ensure food safety [17]. Plant-based beverages containing abundant amounts of phenolic compounds including natural pigments such as anthocyanins, carotenoids and xanthophylls are preferred by consumers compared to synthetic or chemically treated carbonated beverages. There is no literature available on the transformation of purple corn kernels into a value-added PCM beverage. Because of higher bioactivity and antioxidant potential, purple corn or other plant based beverages without addition of chemical additives could show better future prospects as nutraceutical beverages in reducing the incidence of chronic diseases. Therefore, the aim of our study was to evaluate changes in physicochemical properties, functional components, sensory acceptance and shelf-life extension of purple corn milk (PCM) subjected to boiling and steaming processes. The physicochemical and microbial quality of unpasteurized and pasteurized PCM samples extracted from boiled and steamed corn kernels was also investigated. Moreover, physicochemical, microbial and functional qualities of unpasteurized and pasteurized PCM samples were also monitored along with the effect of sucrose addition on the sensory quality attributes during 12 days of storage at 4 °C.

## 2. Materials and Methods

### 2.1. Chemicals and Microbial Media

All microbiological media were of analytical grade. 2, 2-diphenyl-1-picrylhydrazyl (DPPH) Fluka, Burlington, USA), 6-hydroxy-2, 5, 7, 8-tetramethylchromane-2-carboxylic acid (Trolox) (Fluka, Roskilde, Denmark), Folin Ciocalteu reagent (Carlo Erba, Emmendingen, France), gallic acid (Fluka, Barcelona, Spain), methanol 99.9% (Fisher Scientific, Warrington, UK), potassium chloride (KCl) (Ajax Finechem, Auckland, New Zealand), sodium acetate (CH_3_COONa) (Ajax Finechem, New Zealand), sodium chloride (NaCl) (Fisher Chemical, Geel, Belgium), Kovac’s reagent (Himedia, Thane, India), Gram stain reagents (Medic, Pasig, Philippines), sodium carbonate (Ajax Finechem, Seven Hills, Australia), ethanol 95% (QRëC, Auckland, New Zealand), Voges-Proskauer (VP) reagents (Hardy Diagnostics, Santa Maria, CA, USA) and tartaric acid (Ajax Finechem, New Zealand) were used in this study. Microbial media used in the experiments include Lactose broth, *Escherichia coli* broth, Levine’s Eosin-Methylene Blue agar, tryptone (tryptophane) broth, MR-VP broth, Koser’s citrate broth and Brilliant Green Lactose Bile broth (Himedia, India). Plate count and potato dextrose agar media (Sigma Aldrich, St. Louis, MI, USA) were also used.

### 2.2. Procurement and Preparation of Fresh Purple Corn Milk

Purple corns (PC) were purchased from a local fresh market in Bangkok in May 2022 and transported to the Food Analysis Laboratory, Department of Food Technology, Chulalongkorn University. PC were husked and corn silk was isolated manually before washing. PC were washed with sterile water to remove the adhered soil and dust particles. After washing, PC were drained in a stainless steel basket to remove the excess water prior to separation of purple corn kernels (PCK) from the corn cob. PCK were peeled off using a stainless steel handheld corn peeler. PCK were placed in a laminated aluminum bag, sealed with a vacuum sealer (Multivac, A300/16, Germany) and stored at −18 °C. 

PCK were defrosted at room temperature for 30 min. PCK (220 g per sample) were subjected to two thermal unit operations (boiling and steaming) for 5, 10 and 15 min. Steaming was carried out in a stainless steel pot in which the bottom container was filled with water to generate steam by placing it on a cooking gas stove. The temperature of the water was monitored by a digital thermocouple (Digi-Sense, Cole Parmer Instrument Co., Veron Hills, IL, Springfield, USA) and adjusted to 110 °C. PCK samples were placed on a perforated stainless steel pot tray to allow the uniform distribution of steam during 5, 10 and 15 min of treatment time. Boiling of PCK was carried out in a hot-water bath with a preset temperature of 100 °C as monitored by a thermocouple. PCK (220 g per sample) were immersed in the hot-water bath and boiled for 5, 10 or 15 min. Both boiled and steamed PCK samples were spread on a stainless steel screen to remove excess water prior to blending. Thermally treated and untreated PCK samples were blended for 1 min with a kernel to sterile water ratio of 1:2 (*w*/*v*) and passed through a sterile muslin cloth to extract purple corn milk (PCM). 

PCM was extracted from untreated, boiled and steamed PCK samples in three batches, and 200 mL of each sample was filled in amber-colored glass bottles before being sealed with a lug-capping machine (Shanghai Harvest Electronics Co., Ltd. Shanghai, China). The first batch of PCM was made without any treatment of PCK and served as control. The second batch of bottled PCM was made from boiled PCK, without and with pasteurization. Finally, the third batch of PCM was made from steamed PCK, with and without pasteurization. All PCM samples filled in amber-colored glass bottles from the second and third batches were pasteurized for 15 s at 72 °C [18] and immediately cooled in an ice-water bath. Pasteurization was carried out in a water bath, and the central cold-point temperature of PCM bottles was monitored with a digital thermocouple (Digi-Sense, Cole Parmer Instrument Co., Veron Hills, IL, USA). All sample acronyms used in this article are defined below:

- PCM-CON: Purple corn milk sample without any treatment
- PCM-B5, PCM-B10, PCM-B15: Purple corn milk samples without pasteurization, extracted from corn kernels boiled for 5, 10 and 15 min at 100 °C, respectively.
- PPCM-B5, PPCM-B10, PPCM-B15: Purple corn milk samples with pasteurization, extracted from corn kernels boiled for 5, 10 and 15 min at 100 °C, respectively.
- PCM-S5, PCM-S10, PCM-S15: Purple corn milk samples without pasteurization, extracted from corn kernels steamed for 5, 10 and 15 min at 100 °C, respectively.
- PPCM-S5, PPCM-S10, PPCM-S15: Purple corn milk samples with pasteurization, extracted from corn kernels steamed for 5, 10 and 15 min at 100 °C, respectively.

### 2.3. Analysis of Physicochemical, and Functional Quality Changes of Thermally Processed PCM

#### 2.3.1. Color, pH, Total Soluble Solid and Viscosity

Color values of boiled and steamed PCM samples without and with pasteurization treatment were determined with a chroma meter (Minolta, Model CR-300 series, Osaka, Japan) in three replicates (*n* = 3). PCM samples were evaluated for color at room temperature. CIE-Lab values of L* (lightness), a* (redness) and b* (yellowness) were also determined [19]. The pH values and total soluble solid (TSS) of PCM samples were recorded in three replicates (*n* = 3) by a pH meter (Inobab, TetraCon 325, Cologne, Germany) and a hand-held refractometer (Atago No. 3840, Atago, Tokyo, Japan), respectively [20]. A viscometer with an L adapter (Model Viscobasic + L, Fungilab S.A., Barcelona, Spain) was used to measure viscosity of PCM samples in three replicates (*n* = 3) at 50 °C and 100 revolutions per min. Results of all the sample values were presented as centipoise (cP) [21].

#### 2.3.2. Anthocyanin Content 

The pH differential method was employed for analysis of anthocyanin content as described by Yıldız et al. [22]. The differences in the absorbance values of wavelength at pH_1_ (A516–A700) and pH_4.5_ (A516–A700) values were recorded in three replicates (*n* = 3) for all the samples. Absorbance for all the PCM samples was taken in a Multiskan Go Microplate Spectrophotometer reader (Thermo Scientific, Waltham, MA, USA) and the results were expressed as mg/L of the PCM.

#### 2.3.3. Total Phenolic Compound and Antioxidant Activity by 2, 2-diphenyl-1-picrylhydrazyl Assay

The Folin–Ciocalteu method was employed for the determination of total phenolic compound (TPC) [23]. TPC of 100 µL supernatant (PCM sample) was pipetted into a 10 mL volumetric flask, along with 7 mL of distilled water and 500 µL of Folin–Ciocalleu reagent, and kept at room temperature for 5 min. After that, a saturated sodium carbonate solution (1.5 mL) was added and the volume was adjusted to 10 mL with distilled water before incubating for 2 h at room temperature in the dark. In the same way, a standard curve for gallic acid (0–0.5 mg/mL) was created. The absorbance of the samples and the gallic acid solution was then measured in three replicates (*n* = 3) using a spectrophotometer at 765 nm. TPC was measured in milligrams of gallic acid equivalents per 100 mL (mg GAE/100 mL). 

The methods of Brand-Williams et al. [24] was used to assess antioxidant activity using 2, 2-diphenyl-1-picrylhydrazyl (DPPH). A 250 mL PCM sample was mixed with 4.75 mL DPPH methanol solution, vortex-mixed and incubated at room temperature for 15 min in the dark. Similarly, a standard curve for trolox (0–0.5 mg/mL) was created. A spectrophotometer was used to detect the absorbance of DPPH samples in three replicates (*n* = 3) at 515 nm. The following equation was used to calculate antioxidant activity using the DPPH assay following Equation (1):

A_diff_ = A_initial_ − A_final_ (1)

where A_diff_ is the difference of the absorbance between DPPH and sample, A_initial_ is absorbance of DPPH and A_final_ is absorbance of the sample. DPPH was expressed as mM trolox equivalents/100 mL (mM TE/100 mL). 

### 2.4. Preparation of PCM Samples Fortified with Sucrose without and with Pasteurization

A PCM sample extracted after 5 min of steaming at 110 °C was divided into several portions and 2–6% of sucrose was added. An unpasteurized PCM sample obtained from 5 min steamed PCK without sucrose addition served as a control. PCM samples fortified with different sucrose levels were bottled and sealed followed by pasteurization at 72 °C for 15 s [18], and subsequently cooled down in an ice-water bath as described in Section 2.2. All the acronyms of PCM unpasteurized and pasteurized samples without and with sucrose are defined as follows:

PCM5-S0: Unpasteurized PCM extracted from corn kernels after 5 min of steaming at 110 °C without addition of sucrose
PCM5-S2, PCM5-S4, PCM5-S6: Pasteurized PCM samples extracted from kernels after 5 min of steaming at 110 °C and fortified with 2, 4 and 6% of sucrose.

### 2.5. Analysis of Physicochemical, Functional, Microbial and Sensory Quality Retention of PCM with Added Sucrose during Refrigerated Storage

PCM samples extracted from corn kernels steamed for 5 min and with 2 to 6% of sucrose added were analyzed for physicochemical, microbial and sensorial quality changes during storage of 12 days at 4 °C. Quality changes of PCM5-S0, PCM5-S2, PCM5-S4 and PCM5-S6 samples were determined as described in Section 2.2. The PCM5-S0 (without sucrose) and PCM samples with different levels of sucrose added were analyzed for *Escherichia coli* and coliform counts (CFU/mL) every 2-day interval of storage time up to 12 days of refrigerated storage using the ISO/DIS 9308-1 (2012) method [25]. During the storage period of 12 days (every 2 days), PCM samples were serially diluted in 0.85% NaCl, and then pour plate and spread plate techniques were performed in triplicate to determine total viable microorganisms and yeast and mold count, respectively. For total viable microorganisms and yeast and mold counts, respectively, plate count agar and potato dextrose agar were used as medium. The plates were incubated at 37 °C for 48 h (total viable microorganisms) and 30 °C for 48 h (yeast and mold). Total microbial counts were calculated and expressed as log CFU/mL of each sample [18].

Sensory evaluation of unpasteurized and pasteurized PCM extracted from corn kernels steamed for 5 min with 2 to 6% of sucrose added was conducted by recruiting 54 untrained panelists of 22–31 years of age (22 males and 32 females) from the Department of Food Technology, Chulalongkorn University. A sensory scoring test was performed using a 9-point hedonic scale where 9 = like extremely, 7 = like moderately, 5 = neither like or nor dislike, 3 = dislike moderately and 1 = dislike extremely. PCM samples (20 mL) with different sucrose levels were placed into transparent tasting cups and labelled randomly with three-digit numbers. During the sample evaluation, panelists were asked to rinse their mouths. Panelists performed sensory analysis in the sensory evaluation laboratory in environmentally controlled partitioned booths lit by white incandescent light. Sensory scores were given for appearance, odor, viscosity, taste and overall acceptability [26].

### 2.6. Statistical Analysis

A completely randomized design (CRD) was used in this study and experimental results were expressed as the means ± standard deviation (SD) of three replicates (*n* = 3) for each treatment. The data were analyzed using Analysis of Variance (ANOVA), and the means were compared using the Duncan’s Multiple Range Test (DMRT) method at a 95% confidence level using the Statistical Package for the Social Sciences (SPSS) version 22.0 [18].

## 3. Results and Discussion

### 3.1. Impact of Thermal Processing on Physical Properties, Bioactive Compounds and Antioxidant Potential of PCM

The pH, total soluble solid (TSS), color and viscosity values of PCM samples without and with pasteurization extracted from boiled (Table 1) and steamed (Table 2) purple corn kernels (PCK) are reported. The pH of all the PCM samples ranged from 6.5 to 6.8 without any marked difference in all the samples (*p* > 0.05). The ranges of TSS in PCM samples from boiled and steamed kennels were 5.5 to 5.7 and 6.6 to 6.7 °Brix, respectively. TSS was lower in PCM-CON and other boiled samples than in the PCM samples prepared from boiled and steamed PCK (*p* ≤ 0.05). The increment in TSS was evidenced in PCM samples extracted from steamed PCK compared to boiled PCK (*p* ≤ 0.05) (Table 2). Sweet corn subjected to microwave, steam and hot-water-blanching processes were reported to show a decrease in TSS in hot-water blanching due to loss of sugars and other nutrients from immersing the kernels in water [27]. The viscosity values of PCM obtained from boiled and steamed PCK ranged between 76.8 to 136.9 and 79 to 115.8 cP, respectively. The viscosity of PCM samples increased with longer thermal processing time especially in steamed samples (Table 2) compared to the boiled PCM samples (Table 1), while the PCM-CON showed the lowest viscosity value of 28.1 cP (*p* ≤ 0.05). The higher viscosity displayed by PCM extracted from steamed corn kernels could be a result of starch gelatinization in the steaming method rather than the boiling process. Additionally, pasteurization did not affect the pH values among all the PCM samples (*p* > 0.05). Thermal processing of corn juice at 95 °C for 5 min was reported to promote corn starch gelatinization with higher viscosity compared to untreated control [28].

The range of color values for pasteurized and unpasteurized PCM samples, namely L* (59.7 to 62.9 and 53.6 to 58.3, respectively), a* ( 2.7 to 8.2 and 4.3 to 8.1, respectively) and b* (7.8 to 18.3 and 5.1 to 8.4, respectively), are presented in Table 1 and Table 2. Color values of PCM samples extracted from boiled (Table 1) and steamed (Table 2) PCK for a longer exposure time had increased L* values. L* and b* values before and after pasteurization were higher in boiled PCM samples compared to their steamed counterparts (*p* ≤ 0.05). L* and b* values in the PCM-CON sample were in line with the PCM-S5 and PPCM-S5 samples, respectively (*p* > 0.05). However, the a* values decreased in PCM samples boiled and steamed for 10-15 min. The lowest decrease of a* values was noticed in PCM-S5 and PPCM-S5 samples compared to PCM-CON and other samples exposed to longer boiling and steaming times (*p* ≤ 0.05). The decrease in a* values could be due to anthocyanin decomposition over a longer thermal processing time converting it to chalcone, a colorless compound imparting brightness to PCM samples [29]. Thermal processing involving boiling and steaming have been documented to decrease redness (a*) in frozen vegetables compared to controls without any thermal treatment [16]. The higher L * and b * values and lower a* value among the boiled corn samples could be associated with the leaching out of anthocyanins in water [30]. Purple corn cob containing enormous amounts of anthocyanins have been exploited due to their appealing color for pigment extraction and applied as natural colorant to develop functional foods [11,31]. 

**Figure 1:**
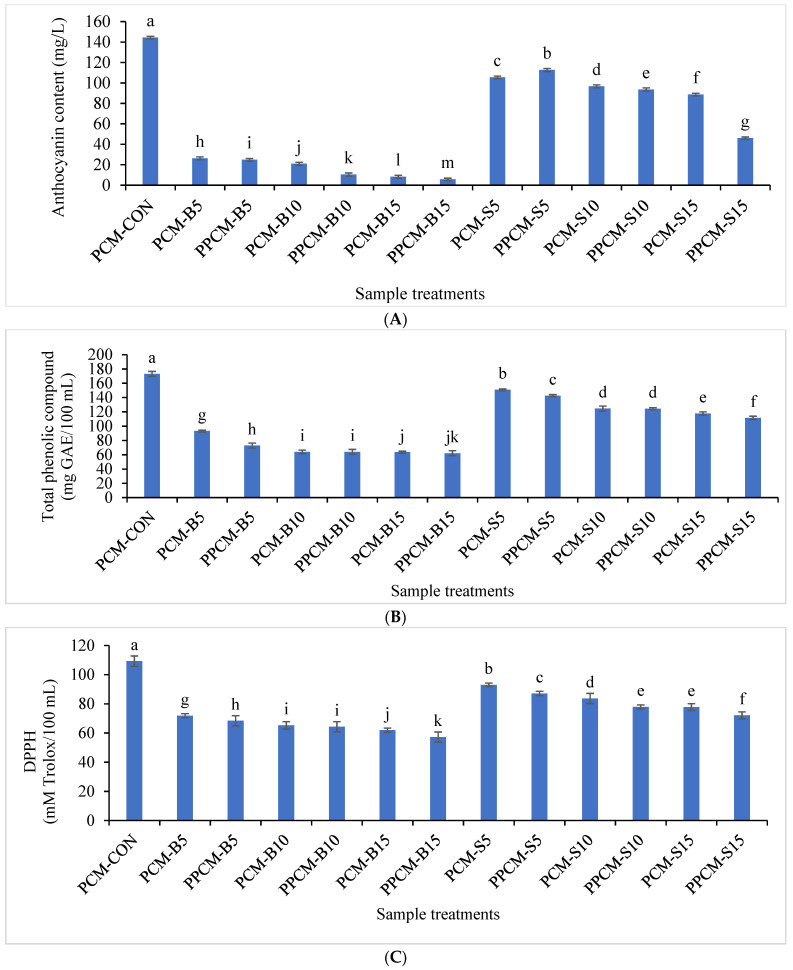
Anthocyanin content (**A**), Total phenolic compound (**B**), and 2,2-diphenylpicrylhydrazyl (DPPH assay) (**C**) of thermally processed PCM. Different lowercase letters on the bars indicate a significant difference (*p* ≤ 0.05). PCM: Purple corn milk; PCM-CON: PCM without any treatment; PCM-B5, PCM-B10, PCM-B15, PPCM-B5, PPCM-B10, PPCM-B15: samples extracted from the boiled corn kernels for 5–15 min without and with pasteurization. Similarly, PCM-S5, PCM-S10, PCM-S15, PPCM-S5, PPCM-S10, PCM-S15: samples extracted from the steamed corn kernels for 5–15 min without and with pasteurization.

The anthocyanin content in PCM extracted from boiled PCK exposed to longer heating time were degraded compared to samples obtained from steamed PCK (*p* ≤ 0.05) (Figure 1A). The PCM-CON sample showed the highest anthocyanin content (*p* ≤ 0.05) compared to the samples prepared from boiled and steamed PCK. However, the highest anthocyanin content was attained in the PPCM-AS5 sample (*p* ≤ 0.05), when compared to the PCM obtained from boiled and steamed PCK. Additionally, the PPCM-B15 sample had the lowest anthocyanin content compared to all the thermally treated PCM samples. In general, thermal treatment times of 5–15 min for both boiling and steaming decreased the amount of anthocyanin in PCM samples (*p* ≤ 0.05). Cyanidin 3-O—D-glucoside was reported as the most abundant anthocyanin followed by pelargonidin 3-O—D-glucoside, peonidin 3-O—D-glucoside, cyanidin 3-O—D-(6-malonyl-glucoside), pelargonidin 3-O—D-(6-malonyl-glucoside), pelargonidin 3-O—D-(6-malonyl-glucoside) and peonidin 3-O—D-(6- (6-malonyl-glucoside) in purple corn [32]. Anthocyanins are degraded into colorless chalcones upon exposure to longer thermal processing times resulting in the fading of the purple color of corn [33]. Steaming of purple-fleshed sweet potatoes (*Ipomoea batatas*) at atmospheric pressure for 30 min was reported to increase anthocyanin content by 40% to retain the purple color [34].

Total phenolic compound (TPC) and antioxidant activity (DPPH) of PCM samples extracted from thermally processed PCK are depicted in Figure 1B,C. TPC of PCM samples extracted from boiled kernels were lower than the PCM samples obtained from steamed kernels (*p* ≤ 0.05). TPC of PCM-CON was highest (*p* ≤ 0.05). The PCM-S5 sample showed the highest TPC amongst the other boiled or steamed samples. The lowest TPC was analyzed in PCM-B15 and PPCM-B15 samples in comparison with the PCM-CON and other thermally treated samples (*p* ≤ 0.05) (Figure 1B). TPC was severely affected in the boiled kernel samples because of longer heating exposure. A similar trend was displayed for the DPPH-radical-scavenging activity in which higher values were attained in PCM-CON followed by PCM-S5 samples compared to the other treated samples (*p* ≤ 0.05). The lowest DPPH values were obtained in PCM-B15 and PPCM-B15 samples than the PCM-CON and samples obtained from PCK after boiling or steaming (*p* ≤ 0.05) (Figure 1C). Increments in the boiling time of PCK revealed lower DPPH activity, possibly due to the degradation of heat-sensitive polyphenols. DPPH-radical-scavenging activity showed a marked decrease in purple corn flour extract that affected antioxidant potential for being exposed to longer thermal processing at temperatures up to 180 °C [35]. The degradation of heat-sensitive bioactive compounds such as total carotenoids, total flavonols and TPC was highest in boiled kale samples, while steamed kale samples potentially retained the bioactive compounds with enhanced antioxidant activity [36].

### 3.2. Quality Retention of PCM Samples Fortified with Sucrose Followed by Pasteurization, Stored for 12 Days at 4 °C

**Figure 2:**
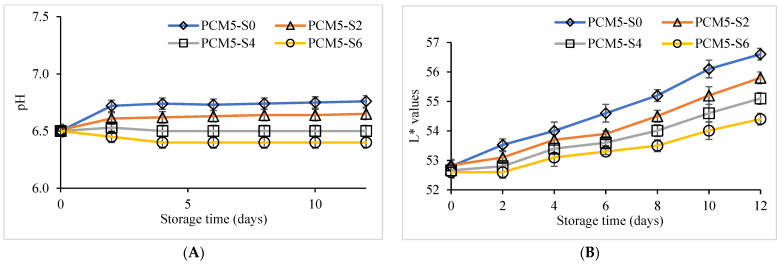

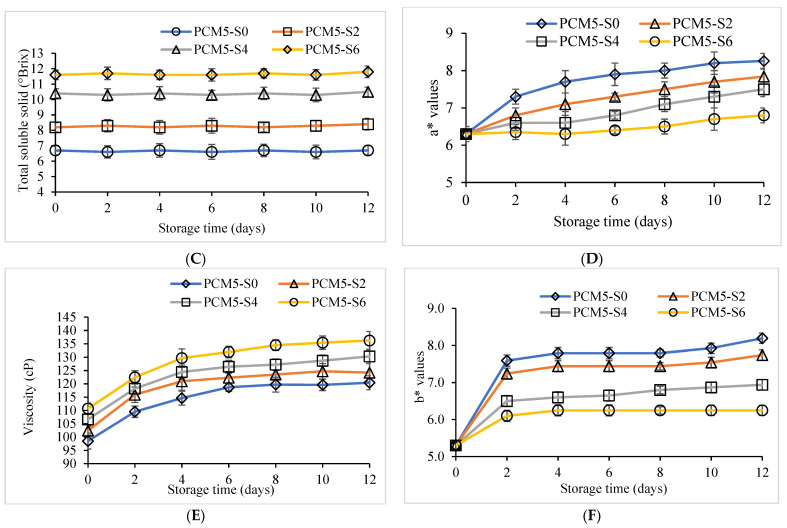
pH (**A**), Total soluble solid (**B**), viscosity (**C**), L* values (**D**), a* values (**E**), and b* values (**F**) of PCM extracted from 5 min steamed purple corn kernels without and with sucrose addition during 12 days of storage at 4 °C. Values are presented as mean ± standard deviation (*n* = 3). PCM: Purple corn milk; PCM5-S0: PCM extracted from 5 min steamed kernels without sucrose addition and pasteurization; PCM5-S2, PCM5-S4, PCM-S6: samples extracted from 5 min steamed kernels fortified with 0 to 6% sucrose followed by pasteurization.

The pH, total soluble solid (TSS) and viscosity values of the PCM samples extracted from 5 min steamed PCK and without and with different sucrose levels added during storage for 12 days at 4 °C are provided in Figure 2. At day 0 of storage, pH values of all the samples were 6.5. As the storage time of PCM samples proceeded, differences in the pH vales of all the samples was noticeable during 12 days at 4 °C. The PCM5-S0 (without pasteurization and sucrose) sample showed a rise in pH compared to the pasteurized PCM5-S2, PCM5-S4 and PCM5-S6 samples during the entire storage period (Figure 2A). The pH of milk was reported to increase during storage due to the degradation of proteins and fatty acids during refrigerated storage [37]. The TSS of PCM samples was in the range of 6.7 to 11.7 °Brix at the first day of refrigerated storage (Figure 2C). With the advancement in storage time up to 12 days, TSS in PCM5-S6 was highest followed by PCM5-S4, compared to PCM5-S2 and PCM5-S0 samples. The PCM5-S0 sample had lower TSS during the entire storage than the samples with different sucrose levels added. Sucrose content in commercially bottled fruit juices was able to main pH and exhibited higher TSS compared to naturally squeezed fruit juices from apple, grapefruit, orange, pineapple, pomegranate, red grape and white grape [38]. 

The viscosity of PCM samples obtained from 5 min steamed PCK without and with sucrose addition was 98.5 to 110.7 cP at day 0 of refrigerated storage (Figure 2E). After day 2 of storage, viscosity of all the samples tended to increase, and the highest viscosity values were noticed in pasteurized PCM5-S6 followed by PCM5-S4 and PCM5-S2 samples in comparison with the unpasteurized PCM5-S0 without sucrose. The PCM5-S0 sample had the lowest viscosity during 12 days of refrigerated storage. The results were in line with the TSS of the aforementioned PCM samples with 4 to 6% of added sucrose concentration (Figure 2C). Additionally, sucrose addition in apple juice was reported to exhibit higher viscosity [34]. Changes in pH, TSS and viscosity directly affects the acceptability of processed liquid foods during storage [35].Color values of PCM samples supplemented with 0 to 6% sucrose content stored at refrigerated conditions for 12 days are presented in Figure 2. The highest color changes in L*, a* and b* values occurred in the unpasteurized PCM5-S0 sample, while the lowest changes in color were recorded in the pasteurized PCM5-S4 sample during 12 days of storage. Lightness of the PCM5-S0, PCM5-S2, PCM5-S4 and PCM5-S6 samples increased based on the initial L* values at day 0 up to 12 days of refrigerated storage (Figure 2B). However, the lowest decease in L* values occurred in the PCM5-S4 and PCM5-S6 samples that could be attributed to the preservation of purple color during storage compared to the unpasteurized PCM5-S0 sample. Additionally, a* and b* values were increased during the refrigerated storage of 12 days in all the samples (Figure 2D,F). However, the highest and lowest increases in a* and b* values were noticed in PCM5-S0 and PCM5-S6 followed by PCM5-SC4 from day 0 to 12 days of storage, respectively. PCM5-S0 without sucrose and pasteurization faded in color during the entire storage, possibly due to degradation of anthocyanins responsible for the purple color. Color is an important factor that directly affects quality of food products and consumer acceptance [36]. Similar reports of anthocyanin degradation were documented in purple vegetables without thermal processing due to active enzymatic reactions [37,38,39,40,41,42]. The color changes are an indicator of freshness or deterioration of food products and may also be induced by enzymatic browning [43].

**Figure 3:**
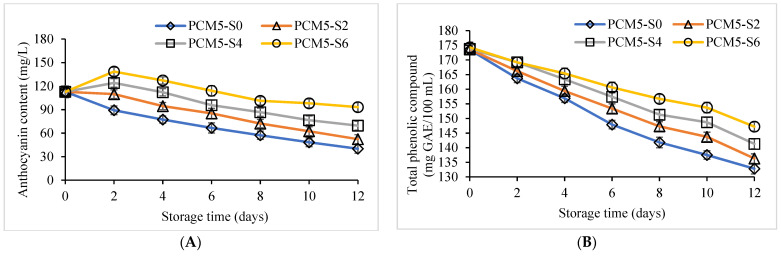

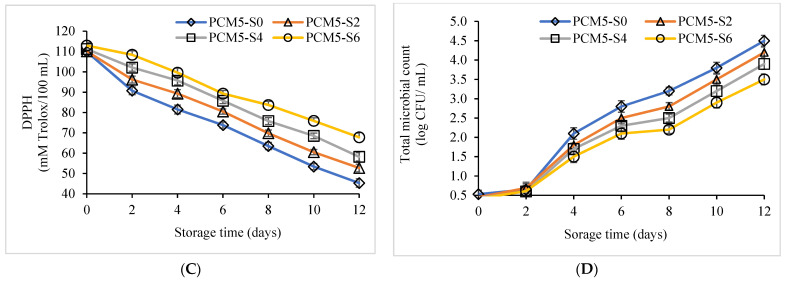
Anthocyanin content (**A**), total phenolic compound (**B**), DPPH (**C**), and total microbial count (**D**) of PCM extracted from 5 min steamed purple corn kernels without and with 0–6% sucrose added during 12 days of storage at 4 °C. Values are presented as mean ± standard deviation (*n* = 3). See Figure 2 caption.

Anthocyanin contents of unpasteurized and pasteurized PCM samples added without and with different sucrose levels are displayed in Figure 3A. The lowest decrease in anthocyanin content occurred in PCM5-S6 followed by PCM5-S4 samples compared to the other samples. PCM5-S0 marked the highest anthocyanin degradation during the entire refrigerated storage of 12 days. On the other hand, TPC was also safeguarded in pasteurized PCM5-S6 followed by PCM5-S4 and PCM5-S2 samples (Figure 3B). The range of TPC in the PCM5-S4 and PCM5-S6 samples was 141.3 to 147.2 mg GAE/ 100 mL compared to PCM5-S0 (132.8 mg GAE/100 mL) samples at day 12 of storage (Figure 3B). A similar trend was observed in the antioxidant activity values assessed with the DPPH method during 12 days of storage. The highest antioxidant activity was determined in PCM5-S6 followed by the PCM5-S4 sample in comparison with the PCM-S0 samples during refrigerated storage of 12 days. The lowest values of DPPH radical-scavenging activity was obtained in PCM5-S0 samples at day 12 (Figure 3C). Anthocyanins in purple corn might be regarded as an important quality attribute that directly affects its acceptability in the consumer market. Anthocyanins have tremendous health benefits and PCM beverage rich in anthocyanins could be labelled as a potential functional product. Purple corn anthocyanins are polyphenolic compounds with natural antioxidant effects with proven facts to supply extra electrons to free radicals, avoiding lipid oxidation and degradation of vital nutrients [44]. Traditional thermal cooking processes in purple waxy corn conducted at 90 °C for 30 min showed lowered anthocyanin, TPC, antioxidant activity [45]. Anthocyanins are a class of natural water-soluble flavonoids with different color variation from blue to purple, and their stability could be influenced by different factors such as pH, light, temperature, co-pigmentation, sulfites, ascorbic acid, oxygen and enzymes [12]. The anthocyanins, TPC and antioxidant activity in waxy purple corn cob extract powder were reported to decrease during 30 days of storage at 4 °C [9]. 

Total viable microbial counts of unpasteurized and pasteurized PCM samples fortified with sucrose during storage of 12 days are presented in Figure 3D. Total microbial load of all the PCM samples was estimated to be 0.4 to 0.5 log CFU/ mL at day 0 of storage. With the progression of storage time from days 2 to 12, microbial counts in PCM samples increased. The highest increase was evidenced in the unpasteurized PCM5-S0 sample versus the pasteurized PCM samples supplemented with 2–6% sucrose. The lowest total viable microbial counts were obtained in PCM5-S6 followed by PCM5-S4 samples compared to that of PCM5-S0. The population of coliforms was less than 1 MPN/100 mL and *Escherichia coli* were not detected in all the PCM samples fortified with sucrose during 12 days of refrigerated storage. *E. coli* contamination in fresh foods upon exposure to human body may induce bacillary dysentery symptoms at a minimum limit of 10 CFU/mL [46]. Additionally, yeast and mold count was also absent during the entire storage period. This clearly depicts that hygiene was maintained and cross-contamination was prevented during preparation of PCM from 5 min steamed PCK. Furthermore, the acceptable microbial quality load for pasteurized beverages was reported to be ≤ 6 log CFU/ mL [47].

Sensory evaluation of unpasteurized and pasteurized PCM samples supplemented without or with different levels of sucrose were evaluated by untrained panelists at day 0 of refrigerated storage (Table 3). At the beginning of storage time (day 0), the highest scores were given to the PCM5-S4 and PCM5-S6 samples based on the appearance, color, odor, taste and overall acceptability compared to PCM5-S0 (*p* ≤ 0.05). However, sensory scores for viscosity and taste were lowest in PCM5-S6 samples at the first day of storage. As the storage time proceeded to 12 days, physicochemical and microbial changes occurred in all the samples (Figure 2 and Figure 3). Changes in anthocyanin content, viscosity and TSS affected the overall sensory attributes of PCM samples during storage. At day 12 of refrigerated storage, the unpasteurized PCM5-S0 sample was discarded due to safety concerns for untrained panelists because it had the highest quality degradation and elevated microbial load. Therefore, higher sensory quality was retained in pasteurized PCM5-S4 compared to PCM5-S6 (*p* > 0.05) regardless of the similarity in color scores at day 12 of storage. The similar scores for color or appearance in PCM5-S4 and PCM5-S6 samples were correlated with the stability of anthocyanin content during 12 days of storage (Figure 3A). Moreover, the PCM sample with 6% sucrose content was given the lowest score by panelists compared to PCM with only 4% sucrose. The shelf-life of pasteurized PCM5-S4 received higher sensory acceptance based on medium sucrose level and retention of overall sensory attributes than the other PCM samples. Purple corn anthocyanins supplemented in milk prevented the oxidative changes of unsaturated fatty acids and preserved the sensory quality during low temperature storage for 7 days [29]. Anthocyanins from purple corn fortified in milk preserved the nutritional, sensory quality and consumer acceptability during 7 days of refrigerated storage [48]. 

## 4. Conclusions

PCM samples prepared from 5–15 min boiled and steamed PCK showed no changes in the pH and TSS values. Viscosity values were higher in PCM samples extracted from boiled PCK than from steamed. Pasteurized PCM (PPCM-S5) samples extracted from PCK steamed for 5 min at 110 °C retained higher anthocyanins compared to PCM-CON and PCM prepared from boiled PCK. Anthocyanins, TPC, and antioxidant activity were retained by 28.3, 21.8 and 25.5%, respectively, in the PPCM-S5 sample in comparison with PCM-CON. Lightness (L*) of the samples was lower in PPCM-S5 than unpasteurized and pasteurized PCM obtained from boiled or steamed PCK. The PPCM-S5 sample showed lower b* values and higher a* values compared to other PCM samples. The pasteurized PCM5-S4 sample fortified with 4% sucrose showed the lowest changes in color, viscosity, pH and TSS during 12 days of refrigerated storage compared to the PCM5-S0 sample. Additionally, the PCM5-S4 sample had the least changes in anthocyanins, TPC and DPPH-radical-scavenging activity during 12 days of storage. Total microbial count was <3.5 log CFU/mL in the PCM5-S4 sample, while *E. coli*, yeast and mold were not detected in all the samples during 12 days of storage. Moreover, the highest sensory scores of appearance, color, viscosity, taste and overall acceptability were attained in the PCM5-S4 sample. Therefore, the shelf-life of PCM5-S4 was extended by 6 days compared to the PCM5-S0 sample, which became unacceptable after 6 days of storage at 4 °C. This work could be beneficial for the beverage industry to develop a low-sucrose value-added plant-based milk product from underutilized purple corn rich in anthocyanins and antioxidants as a functional beverage for consumer acceptance and wellbeing. 

## Figures and Tables

**Table 1:** Physicochemical properties of unpasteurized and pasteurized purple corn milk extracted from boiled purple corn kernels.

Sample Treatments	L*	a*	b*	ΔE	pH	Total Soluble Solid (°Brix)	Viscosity (cP)
PCM-CON	52.6 ± 0.6 ^c^	8.2 ± 0.3 ^a^	7.8 ± 0.6 ^f^		6.5± 0.2 ^a^	5.6 ± 0.4 ^a^	28.1 ± 2.3 ^g^
PCM-B5	59.7 ± 3.2 ^bc^	7.1 ± 0.4 ^b^	12.3 ± 0.5 ^e^	8.47	6.6 ± 0.3 ^a^	5.7 ± 0.2 ^a^	76.8 ± 3.9 ^f^
PPCM-B5	60.5 ± 0.6 ^bc^	6.4 ± 0.3 ^bc^	13.8 ± 0.6 ^d^	10.08	6.7 ± 0.2 ^a^	5.5 ± 0.3 ^a^	88.7 ± 2.9 ^e^
PCM-B10	61.4 ± 0.7 ^b^	5.8 ± 0.2 ^c^	15.1 ± 0.3 ^c^	11.68	6.7 ± 0.2 ^a^	5.5 ± 0.2 ^a^	98.8 ± 2.8 ^d^
PPCM-B10	61.9 ± 0.4 ^b^	4.1 ± 0.4 ^d^	16.5 ± 0.7 ^bc^	13.37	6.7 ± 0.3 ^a^	5.5 ± 0.2 ^a^	105.3 ± 3.9 ^c^
PCM-B15	62.7 ± 0.6 ^a^	3.4 ± 0.2 ^e^	16.8 ± 0.5 ^b^	14.35	6.7 ± 0.2 ^a^	5.5 ± 0.3 ^a^	113.8 ± 2.6 ^b^
PPCM-B15	62.9 ± 0.5 ^a^	2.7 ± 0.2 ^f^	18.3 ± 0.4 ^a^	15.70	6.8 ± 0.3 ^a^	5.5 ± 0.4 ^a^	136.9 ± 3.5 ^a^

Values are mean ± standard deviation (*n* = 3). Different superscripts within the same column followed by different lower case letters (a–g) indicate a significant difference (*p* ≤ 0.05). PCM: Purple corn milk; PCM-CON: PCM without any treatment; PCM-B5, PCM-B10, PCM-B15, PPCM-B5, PPCM-B10, PPCM-B15: samples extracted from the boiled corn kernels for 5–15 min, without and with pasteurization. L* (lightness), a* (redness) and b* (yellowness).

**Table 2:** Physicochemical properties of unpasteurized and pasteurized purple corn milk extracted from steamed purple corn kernels.

Sample Treatments	L*	a*	b*	ΔE	pH	Total Soluble Solid (°Brix)	Viscosity (cP)
PCM-CON	52.6 ± 0.6 ^cd^	8.2 ± 0.3 ^a^	7.8 ± 0.6 ^b^		6.5± 0.2 ^a^	5.6 ± 0.4 ^b^	28.1 ± 2.3 ^f^
PCM-S5	53.6 ± 0.3 ^cd^	8.1 ± 0.3 ^a^	7.4 ± 2.6 ^b^	1.08	6.5± 0.2 ^a^	6.7 ± 0.1 ^a^	79.5 ± 2.1 ^e^
PPCM-S5	52.1 ± 0.4 ^d^	7.2 ± 0.4 ^b^	5.1 ± 0.3 ^c^	2.92	6.6 ± 0.2 ^a^	6.6 ± 0.2 ^a^	98.4 ± 2.9 ^d^
PCM-S10	55.5 ± 0.8 ^bc^	6.5 ± 0.2 ^bc^	7.3 ± 0.4 ^b^	3.39	6.6 ± 0.1 ^a^	6.6 ± 0.3 ^a^	103.3 ± 2.7 ^c^
PPCM-S10	56.4 ± 0.5 ^b^	6.1 ± 0.4 ^bc^	6.5 ± 0.6 ^bc^	4.53	6.7 ± 0.1 ^a^	6.6 ± 0.2 ^a^	107.8 ± 2.3 ^bc^
PCM-S15	57.9 ± 0.7 ^ab^	5.2 ± 0.4 ^c^	8.4 ± 0.8 ^a^	6.11	6.7 ± 0.1 ^a^	6.6 ± 0.2 ^a^	110.4 ± 2.6 ^b^
PPCM-S15	58.3 ± 0.3 ^a^	4.3 ± 0.3 ^d^	6.7 ± 0.3 ^bc^	6.99	6.7± 0.1 ^a^	6.6 ± 0.3 ^a^	115.8 ± 2.4 ^a^

Values are mean ± standard deviation (*n* = 3). Different superscripts within the same column followed by different lower case letters (a–f) indicate a significant difference (*p* ≤ 0.05). PCM: Purple corn milk; PCM-CON: PCM without any treatment; PCM-S5, PCM-S10, PCM-S15, PPCM-S5, PPCM-S10, PCM-S15: samples extracted from the steamed corn kernels for 5–15 min, without and with pasteurization. L* (lightness), a* (redness) and b* (yellowness).

**Table 3:** Sensory evaluation of thermally processed PCM fortified with sucrose during storage at 4 °C.

Storage Time (Days)	Sample Treatments	Appearance	Color	Odor	Viscosity	Taste	Overall Preference
0	PCM5-S0	6.5 ± 0.4 ^Ac^	6.7 ± 0.3 ^Ac^	6.6 ± 0.4 ^Ac^	6.5 ± 0.3 ^Ac^	6.7 ± 0.4 ^Ac^	6.7 ± 0.3 ^Ac^
	PCM5-S2	7.6 ± 0.3 ^Ab^	7 ± 0.4 ^Ab^	7.7 ± 0.2 ^Ab^	7.5 ± 0.3 ^Ab^	7.3 ± 0.3 ^Ab^	7.6 ± 0.2 ^Ab^
	PCM5-S4	8.5 ± 0.2 ^Aa^	8.6. ± 0.2 ^Aa^	8.4 ± 0.3 ^Aa^	8.2 ± 0.2 ^Aa^	8.5 ± 0.3 ^Aa^	8.6 ± 0.4 ^Aa^
	PCM5-S6	8.5 ± 0.3 ^Aa^	8.3 ± 0.2 ^Aa^	7.5 ± 0.2 ^Ab^	7.1 ± 0.4 ^Ab^	7.2 ± 0.3 ^Ab^	7.6 ± 0.4 ^Ab^
12	PCM5-S0	-	-	-	-	-	-
	PCM5-S2	5.4 ± 0.4 ^Bb^	5.6 ± 0.2 ^Bb^	5.6 ± 0.3 ^Bb^	5.7 ± 0.3 ^Bb^	5.3 ± 0.3 ^Bb^	5.6 ± 0.2 ^Bb^
	PCM5-S4	6.7 ± 0.2 ^Ba^	6.8 ± 0.2 ^Ba^	6.3 ± 0.2 ^Ba^	6.1 ± 0.2 ^Ba^	6.4 ± 0.2 ^Ba^	6.8 ± 0.3 ^Ba^
	PCM5-S6	6.3 ± 0.3 ^Ba^	6.5 ± 0.3 ^Ba^	5.3 ± 0.4 ^Bb^	5.2 ± 0.3 ^Bb^	5.4 ± 0.4 ^Bb^	5.5 ± 0.4 ^Bb^

Values are mean ± standard deviation (*n* = 54). Different superscripts of uppercase letters (A,B) in the same column within the same treatment and lowercase letters (a–c) in the same column within the same storage time indicate a significant difference (*p* ≤ 0.05). PCM: Purple corn milk; PCM5-S0: PCM extracted from 5 min steamed kernels without sucrose addition and pasteurization; PCM5-S2, PCM5-S4, PCM-S6: samples extracted from 5 min steamed kernels fortified with 0–6% sucrose followed by pasteurization.

## Data Availability

The data presented in this study are available on request from the corresponding author.
